# St. George's Hospital

**Published:** 1915-02-20

**Authors:** 


### St. George's Hospital.

### REVISION OF THE LAWS.

AT the conclusion of the ordinary business of the Court of Governors of St. George's held on Thursday, 11th inst., under the presidency of the Right Hon. Sir Gerald Lowther, the court was made special for the purpose of receiving the report of the Committee on the Revision of the Laws.

The Revision Committee, which was appointed by the House Committee in pursuance of the order of the Court of Governors of February 12, 1914 (see THE HOSPITAL, February 21, 1914, page 563), consisted of the Deputy-Treasurer, Mr. F. J. Frankau, Dr. Cahill, Mr. W. Ward Cook, and Mr. H. S. Pendlebury. The report acknowledges the assistance which the Committee has derived from the co-operation of Dr. J. Nachbar, the hospital superintendent.

Mr. Frankau, who took charge of the submission of the proposals to the court, first moved that the report be taken as read, and then that it be received. Mr. W. C. Bull, F.R.C.S., seconded both propositions and they were approved. Consideration was then given to the report by clauses. The alterations were submitted in each case by Mr. Frankau, seconded by Mr. Bull, and were all adopted.

The court then specifically sanctioned a number of small —mostly verbal—alterations for the consistency of all the regulations on the principles already approved and carried the whole of the recommendations *en bloc*, the President, in submitting the motion, expressing the gratitude of the court to the Committee for their successful labours and the hope that the alterations would prove to be for the benefit of the hospital.

The amended laws are to be substituted for the present ones on May 1, 1915, but without prejudice to the existing rights both of the hospital and of any officer appointed under the present laws.

We hope to consider the effect of the changes at a later date.

